# Metabolic Plasticity in Chemotherapy Resistance

**DOI:** 10.3389/fonc.2020.00281

**Published:** 2020-03-06

**Authors:** Maria Andrea Desbats, Isabella Giacomini, Tommaso Prayer-Galetti, Monica Montopoli

**Affiliations:** ^1^Department of Medicine, University of Padova, Padova, Italy; ^2^Veneto Institute of Molecular Medicine, Padova, Italy; ^3^Department of Pharmaceutical and Pharmacological Sciences, University of Padova, Padova, Italy; ^4^U.O.C. Urology, Azienda Ospedaliera di Padova, Padova, Italy

**Keywords:** cancer, metabolic reprogramming, TCA cycle, Warburg effect, metabolic vulnerabilities, chemoresistance

## Abstract

Resistance of cancer cells to chemotherapy is the first cause of cancer-associated death. Thus, new strategies to deal with the evasion of drug response and to improve clinical outcomes are needed. Genetic and epigenetic mechanisms associated with uncontrolled cell growth result in metabolism reprogramming. Cancer cells enhance anabolic pathways and acquire the ability to use different carbon sources besides glucose. An oxygen and nutrient-poor tumor microenvironment determines metabolic interactions among normal cells, cancer cells and the immune system giving rise to metabolically heterogeneous tumors which will partially respond to metabolic therapy. Here we go into the best-known cancer metabolic profiles and discuss several studies that reported tumors sensitization to chemotherapy by modulating metabolic pathways. Uncovering metabolic dependencies across different chemotherapy treatments could help to rationalize the use of metabolic modulators to overcome therapy resistance.

## Metabolic Reprogramming

The metabolic program between non-proliferating and proliferating cells is different. Non-proliferating cells rely mostly on catabolic reactions while proliferating cells must balance catabolic and anabolic reactions required to sustain enhanced cellular growth (1–3). In normally proliferating cells most **ATP** from glucose is obtained by glycolisis, tricarboxylic acid cycle (**TCA**) and oxidative phosphorylation (**OxPhos)**, while nucleotides, aminoacids, and lipids are provided by intermediate metabolites of these pathways; such as acetyl-CoA for fatty acids, glycolytic intermediates for non-essential aminoacids, and ribose for nucleotides. Tumor cells are characterized by metabolic hallmarks similar to highly proliferating normal cells but, in addition, they develop a high plasticity to metabolic rewiring to sustain enhanced cellular growth in changing microenvironmental conditions (4).

Back in the 1920's, Otto Warburg observed that many tumors depended on glycolysis as the sole source of ATP; even in the presence of oxygen (aerobic glycolysis) (5). Accordingly, the rate of glucose entry to cancer cells was found 20-to-30- fold higher than in normal cells (6), and glucose transporters and key glycolytic enzymes were heavily upregulated (7). Cancer cells under hypoxia induce pyruvate dehydrogenase kinase (**PDK**) that inactivates pyruvate dehydrogenase (**PDH**) (8). Thus, most glucose-derived pyruvate does not enter the TCA cycle and is converted in lactate by the action of lactate dehydrogenase (**LDH**) (9). This is because most tumors produce great quantities of lactate, which is very striking, since glycolysis produces only 2 ATP molecules for each glucose, while oxidative phosphorylation between 30 and 32 ATPs.

Later it became clear that in cancer cells glucose is consumed mainly to supply glycolitic intermediates for anabolic pathways. Glucose-6-phosphate can be oxidized by glucose-6-phosphate dehydrogenase (**G6PD**) to produce reduced nicotinamide adenine dinucleotide phosphate (**NADPH**) and ribose-5-phosphate (**R5P**) through the pentose phosphate pathway (**PPP**). **NADPH** and **R5P** are required for nucleotide synthesis, but also to sustain biosynthetic reactions and to maintain the redox capacity of the cell (1). Moreover, 3-phosphoglycerate could serve as a precursor for serine and glycine metabolism through the one-carbon cycle (10). Pyruvate instead that can be converted into alanine by alanine aminotransferase (**ALT**) (11). In turn, these aminoacids can be metabolized for nucleotide synthesis, DNA methylation, glutathione production and **NADPH** generation (12). Interestingly, several **PPP** enzymes and 3-phosphoglycerate dehydrogenase (**PHGDH**) were found upregulated in some cancer (13–16).

Unlike originally thought, aerobic glycolysis in cancer cells is not a sign of defective oxidative phosphorylation. Instead, high rates of glycolysis inhibit mitochondrial respiration, a phenomenon termed the “Crabtree effect” (17). Indeed, mitochondria function is essential for cancer cell proliferation (18). Mitochondrial redox homeostasis is crucial for maintaining cellular aspartate levels critical for nucleotide synthesis (19). Indeed, aspartate was shown essential for *in vivo* tumor growth (20).

Of great significance, cancer cells require **TCA** cycle intermediates for biosynthetic pathways and **NADPH** production (21). The **TCA** cycle generates citrate that can be exported to the cytosol through the mitochondrial tricarboxylate carrier (**SLC25A1**) to be converted into acetyl-CoA and oxaloacetate by ATP citrate lyase (**ACLY**). (22). Acetyl-CoA can either be employed for fatty acid and cholesterol synthesis (to support membrane biogenesis) or used for protein acetylation reactions, which regulate nuclear transcription as well as cytoplasmic processes like autophagy (23). The **TCA** cycle also provides metabolic precursors for the synthesis of non-essential amino acids, such as aspartate and asparagine from oxaloacetate, or proline, arginine and glutamate from α-ketoglutarate. To cope with the continuous efflux of intermediates cancer cells replenish the **TCA** cycle by increasing or developing the ability to use various carbon sources; including glutamine, acetate, lactate, serine, and glycine (24–27). In particular, tumor cells consume great quantities of aminoacids.

Glutamine is the major contributor of **TCA** intermediates in many cancer cell lines (28). Glutamine is transported into the cell through plasma membrane transporters, like **SLC1A5** (**ASCT2**) and **SLC7A5** (29) and converted into glutamate by glutaminase (**GLS**). Then glutamate is transformed into α-ketoglutarate, by either glutamate dehydrogenase (**GDH**) or transaminases; and α-ketoglutarate enters the **TCA** cycle to maintain the production of citrate. Glutamine can also be directly converted into citrate by reductive carboxylation. The reductive carboxylation of α-ketoglutarate by the inverse reaction of isocitrate dehydrogenase (**IDH**) generates citrate (30). Glutamine reductive carboxylation is particularly important in tumor cells under hypoxic conditions or when mitochondrial respiration is impaired (31). Moreover, **GLS** and **GDH** are upregulated in a wide variety of tumors and its inhibition has been shown to diminish tumorigenesis (32, 33).

Another contributor of **TCA** intermediates is lactate. Some cancer cells can use lactate produced by aerobic glycolysis as a source of energy. More than 50% of the total **TCA** cycle intermediates in breast cancer cells under glucose deprivation derived from lactate (34). Moreover, overexpression of lactate transporters (**MCTs**) is a common finding in some cancers (35).

Enhanced glycolisis and glutamine metabolism in cancer cells support the increase of *de novo* fatty acids synthesis (36). Fast-proliferating cancer cells use fatty acids and cholesterol for biosynthesis of cell membranes, cell signaling and secondary messengers (37), as well as for lipid catabolism through fatty acid β-oxidation (**FAO**) during nutrient deprivation (38). In some cancers such us prostate cancer and lymphoma, lipid-dependent metabolism becomes essential for energy production (39). In physiological conditions, lipid synthesis is restricted to specialized tissues, such as the liver and adipose tissues. Normal cells uptake lipids from the bloodstream, while cancer cells could obtain lipids and lipoproteins exogenously or by *de novo* synthesis (38). A wide variety of tumors have increased expression of crucial lipogenic enzymes such us **ACLY**, acetyl-CoA-carboxylase (**ACC**), fatty acid synthase (**FASN**) (38, 40, 41); as well as present an increase in the transcriptional activities of the sterol regulatory element-binding proteins (**SREBPs**) (42, 43). The upregulation of lipogenic enzymes seems required for tumor progression (40). Interesstingly, some cancer cells harbor adipocyte characteristics like storing excess lipids in lipid droplets (**LD**) (44). **LD** are intracellular storage organelles of neutral lipids mainly found in adipose tissue, but observed in several cell types and tissues (45, 46). **LDs** are dynamic, and their accumulation seem to confer survival advantages to cancer cells (47). Drugs that specifically target **LD** formation are thought to hold greater therapeutic potential compared with general lipid biosynthesis inhibitors (48, 49).

Enhanced glycolisis, glutamine metabolism and fatty acids synthesis are features shared by many cancer cell lines. However, the metabolic phenotype of the tumor *in vivo* is highly heterogeneous, resulting from the combination of intrinsic (genetic and epigenetic changes, tissue of origin, state of differentiation) and extrinsic (oxygen and nutrient availability, metabolic interactions within the tumor microenvironment) factors (50).

## Role of Oncogenes and Tumor Suppressor Genes in Metabolism Reprogramming

One of the intrinsic factors that determine the tumor metabolic phenotype is the activation of oncogenes or deactivation of tumor suppressor genes which result in a metabolic rewiring (51). Tumor metabolism is distinct in tumors harboring different oncogenic alterations. Oncogenes such as RAS, MYC, or PI3K, favor glycolysis over oxidative phosphorylation; whereas tumor suppressors such as p53, PTEN, Von Hippel–Lindau (VHL), or liver kinase B1 (LKB1) have the opposite effect (52).

In particular, MYC expression could activate the pentose phosphate pathway, purine/pyrimidine synthesis and fatty acid oxidation under chemotherapy and radiotherapy (53). MYC directly regulates several glycolytic enzymes such as glucose transporter 1 (**GLUT1**), hexokinase 2 (**HK2**), phosphofructokinase muscle type (**PFKM**), and enolase 1 (**ENO1**); as well as lactate dehydrogenase A (**LDHA**) (54). Moreover, MYC expression increases glutamine uptake and glutaminolysis (55, 56) by inducing the expression of glutamine transporters **SLC1A5** and **SLC7A5** and by repressing the transcription of microRNA-23a/b which targets glutaminase 1 (**GLS1**) (56, 57).

p53 can directly or indirectly influence the expression of genes involved in glucose, **OxPhos** and lipid metabolism, among other pathways (58). p53 inhibits glycolysis dowregulating **GLUTs** and the glycolytic enzyme phosphoglycerate mutase (**PGAM**) (59, 60). p53 also induce the expression of TIGAR (TP53-induced glycolysis and apoptosis regulator), which indirectly inhibits phosphofructose kinase 1 (**PFK1**) diverting glycolytic intermediates into the **PPP** (61). p53 decreases fatty acid synthesis by also inhibiting the **PPP**. p53 directly binds and inhibits **G6PD** leading to decreased production of **NADPH** (14). Moreover, p53 directly repress the expression of SREBP-1 which regulates the expression of fatty acid synthesis enzymes (62). On the other hand p53 enhances fatty acid oxidation. p53 induces two important enzymes involved in fatty acid oxidation, Lipin 1 and carnitine palmitoyltransferase (**CPT1C**) (63, 64). p53 was also reported to transcriptionally induce malonyl-CoA decarboxylase (**MCD**), which catalyzes the conversion of malonyl-CoA to acetyl-CoA, to promote fatty acid oxidation and prevent lipid accumulation (65). p53 enhances mitochondrial **OxPhos** by inducing the expression of the cytochrome c oxidase (COX, complex IV) assembly factor SCO2 (66) or the expression of AIF; which maintains the integrity of mitochondrial NADH:ubiquinone oxidoreductase (complex I) (67). In addition, p53 promotes **OxPhos** by repressing the transcription of pyruvate dehydrogenase kinase 2 (**PDK2**), which inhibits **PDH** (68). **PDK2** repression activates **PDH** reducing the conversion of pyruvate to lactate and increasing the conversion of pyruvate to acetyl-CoA (68).

RAS can influence the glycolytic metabolism through the PI3K-mTOR pathway, or by upregulating glucose flux through hexosamine biosynthesis pathway (**HBP**) or the **PPP** (53). In addition, mutant KRAS activated lipogenesis through induction of **FAS** (69). In a Kras-driven mutant model of spontaneous lung tumorigenesis the uptake and utilization of branched-chain amino acids (**BCAAs**) such as leucine and valine, were increased, as well as the expression of the enzymes responsible for their catabolism (70).

PTEN decreases glycolysis and promotes oxidative phosphorylation. MEFs from PTEN tg mice present high levels of peroxisome proliferator-activated receptor g coactivator-1α (**PGC-1α**), increase the number of mitochondria, increment the levels of oxygen consumption and ATP production, and diminish lactate secretion. Moreover, PTEN decreases the levels of pyruvate kinase isozyme M2 (**PKM2**) and 6- phosphofructo-1-kinase/fructose-2,6-biphosphatase isoform 3 (**PFKFB3**); and elicits the inhibition of the pro-tumorigenic glutaminase **GLS1** thus contributing to the cancer-protection (71).

Of note, most studies on the role of oncogenes/tumor suppressor genes in metabolic reprogramming were performed using cell models with single genetic modifications. It's hard to translate this findings to the tumor *in vivo* which harbors many genetic defects, and whose metabolic profile will depend on their combination.

## Metabolic Heterogeneity in Tumors

Based on the metabolic strategies prioritized by several solid cancers Lehuede et al. (72) proposed a classification of cancer-specific metabolic phenotypes in glycolytic and oxidative tumors. While lung, liver, colorectal cancers, and leukemias rely mostly on glycolysis; lymphomas, melanomas, and glioblastomas behave as oxidative tumors (72). However, there is not a uniform metabolic phenotype across tumors with a similar genetic defect in different organs or genetically different tumors in the same organ (73). A large study of metabolic features in 180 patient-derived melanoma xenografts excluded a general metabolomic signature (74).

Cancer cells reside in poor oxygen and nutrition environments and hence attempt to reprogram the preexisting tissue metabolism for survival (75). The fact that some regions of the tumor could have more access to oxygen or various carbon sources than others (73) explains why tumor cells are metabolically heterogeneous. Intra-tumoral metabolic heterogeneity is maintained through coupled metabolic interactions between distinct cell populations coexisting in the tumor microenvironment. Stromal and tumor cells can compete or alternatively form symbiotic relationships where the metabolic products of a population become a nutrient of another (76).

Tumor cells can promote a “Reverse Warburg effect” in neighboring Cancer-associated fibroblasts (**CAFs**) (77). **CAFs** are stromal cells which often dominate the tumor microenvironment. Reactive oxygen species (**ROS**) produced by cancer cells activates HIF-1α and NFkB in **CAFs** inducing the production and release of energy-rich metabolites as lactate. Cancer cells could in turn take up lactate to fuel mitochondria respiration for energy production and anabolic metabolism (78, 79). This metabolic symbiosis may be controlled by the differential expression of lactate monocarboxylate transporters **MCT1** and **MCT4**. Lactate is released from **CAFs** by **MCT4** and taken up by **MCT1** in cancer cells. Indeed, lactate transporters inhibition reduces lactate uptake, induces a switch to glycolysis, and blocks metabolic symbiosis and tumor progression (80). Interestingly, higher levels of **MCT1** confer a higher metastatic potential to melanoma cells as metastasizing cells depend on **MCT1** to manage oxidative stress (81).

Cancer-associated adipocytes (**CAAs**) are adipocytes infiltrated into the tumor tissue (82). They provide carbon sources, growth factors, and cytokines affecting tumor growth, metastasis, and drug responses (83). **CAAs** frequently release fibroblast growth factors like (FGFs), leptin, adiponectin, IL-1β, IL-6, TNF-α, CCL2, and CCL5; while cancer cells produce signaling molecules to trigger adipocyte lipolysis (84). In the presence of **CAAs** some cancer cells can acquire exogenous free fatty acids (**FFAs**) released by **CAAs** through the cell surface fatty acid translocase **CD36** and switch their metabolic program from glycolysis to **FAO** (38). *In vitro*, ovarian cancer cells induce white adipocytes lipolysis, fatty acids uptake and **FAO** (85).

Tumor metabolism also modulates the activity of tumor-associated immune populations. Activated T cells and cancer cells share some metabolic similarities (86) giving rise to a competition for nutrients which could impair the immune function (87). Naive CD4 T cells use mitochondrial **OxPhos** as a primary energy source, but upon activation they increase the expression of **GLUT1** and shift to aerobic glycolysis (88). Also TAMs Tumor Associated Macrophages (**TAMs**) **M1** rely on glycolysis to meet increased energetic demands (89). Moreover, increased lactate levels due to enhanced tumoral glycolisis can lead to diminished antitumour immunity. Lactate inhibits FIP200, leading to defective autophagy and increased apoptosis of naive T cells (90). Lactate can also suppress NK and dendritic cell function but does not affect regulatory T (**Treg**) cell function (91). Lactate could also induce the conversion of **M1** to **M2** pro-tumoral macrophages (92). CD8+ T cells and NK cells, are also sensitive to glutamine, serine, glycine, leucine, isoleucine and valine aminoacid restriction (93, 94). Moreover, limited availability of extracellular glutamine shifted the balance from Th1 to Treg cells (95).

Some cancer cells harbor an “hybrid glycolysis/ **OxPhos** phenotype” which allows them to use both glycolysis and the byproducts from glycolysis by **OxPhos** for energy production and biomass synthesis (96). Metabolic plasticity may be specifically associated with metastasis and therapy-resistance because a hybrid metabolism could maintain low **ROS** levels which induce a moderate stress response and the appearance of mutations that further stimulate tumorigenesis and metastasis (97). Dual inhibition of glycolysis (by 2-Deoxy-d-glucose, 2-DG) and **OxPhos** (by metformin) has been shown to effectively repress tumor growth and metastasis across multiple preclinical cancer models (98). Thus, a combination of glycolytic and **OxPhos** inhibitors could effectively eliminate the tumor survival potential of hybrid cells (99).

**Figure 1:**
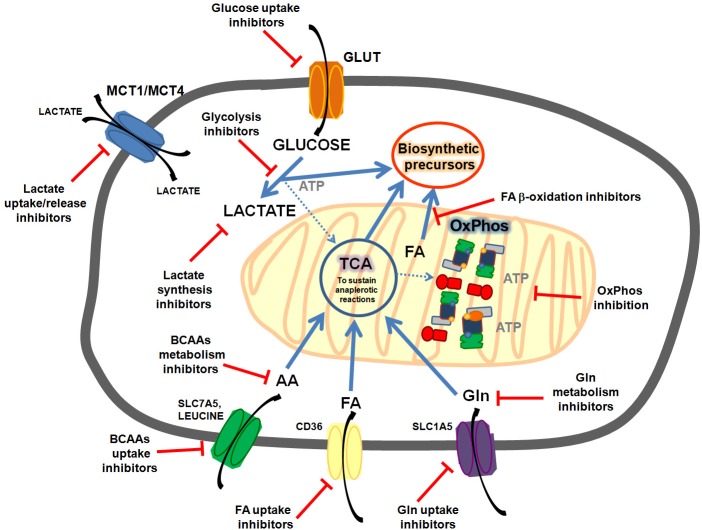
Common metabolic features targeted in cancer cells. Cancer cells could present enhanced glycolisis and lactate release, enhanced FA (fatty acids) synthesis, uptake and oxidation; enhanced OxPhos activity, enhanced glutamine uptake and metabolism, enhanced branched amino acids (BCAAs) uptake and oxidation, etc. Targeting these pathways could sensitize cancer cells to chemotherapy.

Understanding the factors that influence tumor heterogeneity is fundamental for the development of therapies that could act modulating tumor metabolism. Up to now tumor heterogeneity and toxicity issues has limited the success of most clinical trials targeting metabolic pathways (Figure 1).

## Targeting Cancer Metabolism to Overcome Drug Resistance

Recently, metabolic reprogramming has been shown to play a role in the response of cancer cells to widely-used first-line chemotherapeutics (100).

Chemotherapeutic drugs target a differential feature of cancer cells that help them to actively proliferate. The main types of chemotherapy agents used in the clinics are: alkylating agents and platinants (damage DNA), such as cisplatin (101); cytotoxic antibiotics (bind DNA to prevent DNA and/or RNA synthesis); inhibitors of topoisomerase (damage DNA), such as daunorubicin, doxorubicin, irinotecan and etoposide; antimetabolites (interfere with intermediary metabolism of proliferating cells), such as gemcitabine; anti-microtubule agents (target microtubules and associated proteins required in cell division), such as paclitaxel and docetaxel (102); hormonal agents (inhibit hormone synthesis or function as hormone receptor agonist/antagonist) (103), such as tamoxifen or enzalutamide; and immunotherapy (target cancer cells that express a specific antigen or boost the natural ability of T cells to fight cancer), such as trastuzumab.

Tumor recurrence results from the ability of specific tumor subpopulations to resist treatment and expand. Resistance constitutes a lack of response to drug-induced tumor growth inhibition and it may be inherent to a subpopulation of cancer cells or can be acquired as a consequence of drug exposure. Chemoresistance is caused through genetic mutations in various proteins involved in cellular mechanisms such as cell cycle, apoptosis and cell adhesion (104). Reported chemoresistance mechanisms include: altered drug membrane transport, mutation, increased expression of drug targets, decreased drug activation, increased drug degradation due to altered expression of drug-metabolizing enzymes, drug inactivation due to conjugation with glutathione, altered drug subcellular redistribution, drug interactions, enhanced DNA repair, overexpression of anti-apoptotic genes, inactivation of apoptotic gene products, among others (105).

**Figure 2:**
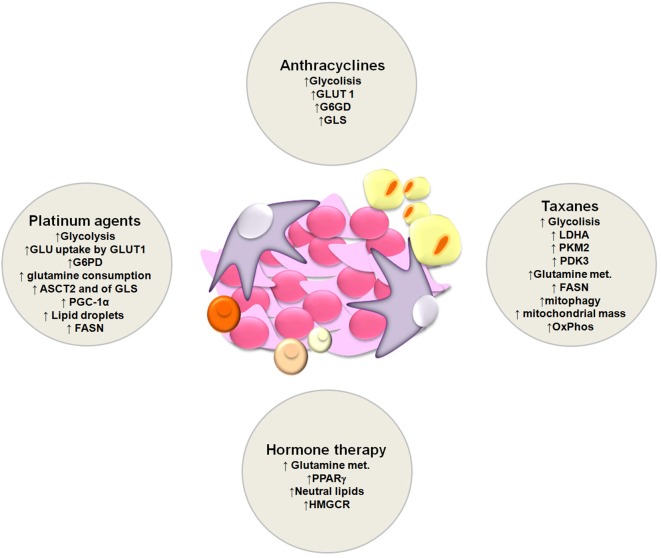
Schematic representation of metabolic alterations involved in the onset of resistance to platinum agents, anthracyclines, taxanes and hormone therapy.

In the last decades, several studies have demonstrated that metabolic reprogramming plays an important role in the onset of chemotherapy resistance (106). Mostly due to the fact that chemotherapy agents used in the clinics cause a compensatory metabolic reprogramming in cancer cells (Figure 2). Thus, implementation of combinatorial therapies with chemotherapeutic drugs and metabolic modulators (Table 1) might provide a way to overcome therapy resistance.

**Table 1:** Overview of promising combination therapy of chemotherapeutic agents and metabolic modulators.

**Chemotherapeutic agents**	**Targets**	**Therapeutic strategy**	**Type of cancer**	**References**
Cisplatin	- Increased expression and enzymatic activity of G6PD	Combination of 6-AN and CDDP	Ovarian cancer	(107)
	- Increased glutamine consumption and increased expression of the glutamine transporter ASCT2 and of GLS	Combination of BPTES and CDDP	Ovarian cancer	(108)
	- Increased PGC-1α levels	Silencing PGC-1α	Small cell lung carcinoma	(109)
	- Increased expression of FASN	Combination between Orlistat and CDDP	Lung carcinoma	(110)
Doxorubicin	- Increased expression of 6- phosphogluconate dehydrogenase (G6GD)	G6GD knockdown or Physcion treatment	Anaplastic thyroid cancer	(111)
	- Increased glycolysis	3-bromopyruvate treatment	Neuroblastoma	(112)
Daunorubicin	- Increased GLUT1 expression	Combination between phloretin and daunorubicin	Leukemia cancer	(113)
Paclitaxel	- Increased expression and activity of LDHA	Downregulation of LDHA or Oxamate treatment	Breast cancer	(114)
	- Increased glycolisis	Combination of 2-DG and paclitaxel	Human osteosarcoma and non-small cell lung cancer	(115)
Docetaxel	- High fatty acid synthase (FASN) activity	Developing	ErbB2-induced breast cancer	(116)
	- Shift from glycolysis toward OXPHOS	Combination of docetaxel and OXPHOS inhibitors	Prostate cancer	(117)
	- mtDNA depletion	Developing	Prostate cancer	(118)
	- Increased mitochondrial mass	Developing	Breast cancer	(119, 120)
Tamoxifen	- Increased level of neutral lipids, in particular, cholesterol esters and triglycerides	Developing	Breast cancer	(121)
	- Increased expression of Peroxisome Proliferator-Activated Receptor Gamma (PPARgamma)	Developing		
Enzalutamide	- Increased expression of HMGCR	Combination of simvastatin and enzalutamide	Prostate cancer	(122)

## Targeting Glucose Metabolism

As we already mentioned above, intensive aerobic glycolysis generates the accumulation of lactate that results in acidosis and promotes tumor progression and metastasis by inducing immunosuppression, vascularization, aggressive proliferation, migration, invasion and therapy resistance (123, 124). It has been demonstrated that enhanced glucose uptake and improved aerobic glycolysis are capable to induce the intrinsic or acquired resistance to chemotherapy in several tumor cells such as breast (125), or ovarian (107). Several key glycolytic enzymes and glucose transporters inhibitors are currently in preclinical or clinical development to counteract resistance to chemotherapeutic drugs (100, 107, 126–129).

Some reports proposed that aerobic glycolysis is an important pathway for colorectal cancer (CRC) development. In fact, the overexpression of the immune checkpoint protein B7-H3 in CRC cells enhanced glucose consumption and lactate release by **HK2** expression, while B7-H3 knockdown had the opposite effect. Moreover, it is known that the depletion of **HK2** expression or **HK2** inhibition blocked aerobic glycolysis and CRC chemo-resistance (130). Recent studies reported that human colorectal adenocarcinoma doxorubicin-resistant cells (LoVo DOX) presents over expression of **GLUT1**. Thus, the treatment with silybin (a modulator of **GLUTs**) resulted synergic with the chemotherapeutic agents and it was able to overcome doxorubicin resistance (131).

Several key glycolytic enzymes, comprising **HK2**, **PFK**, and **PKM2**, are highly expressed in ovarian cancer cells and were implicated in anti-apoptotic and cell survival processes which correlate with chemo-resistance. These enzymes are controlled by oncogenes (e.g., Akt, mTOR) and tumor suppressors (e.g., p53) that may drive deregulated metabolism and ovarian cancer development (132). A few publications reported that ovarian cell lines with high glycolysis rate also presented high **OxPhos** activity, showing that most ovarian tumor cell lines prefer a highly glycolytic metabolic phenotype (133). Several inhibitors of glycolysis, such as **2-DG**, 3-bromopyruvate (**3-BrPA**) or lonidamide (**LND**), have been studied in recent years. The combined treatment between metformin and **2-DG** decreased the cellular growth of ovarian cancer cells (134). Moreover, **2-DG** was able to sensitize cisplatin (**CDDP**)-resistant and radio-resistant cervical CaSki cell lines (135). **3-BrPA** (pyruvate analog) is an inhibitor of **HK2** and an alkylating agent. **LND** is another inhibitor of **HK2**, that enhanced **CDDP** activity in ovarian cancer cells (136). **LND** in combination with the chemotherapeutic agents, **CDDP**, and paclitaxel, presented a good activity and tolerability (137). The **PPP** is another pathway involved in ovarian drug resistance. It was demonstrated that ovarian **CDDP**-resistant cells (C13) showed increased glucose uptake, the up-regulation of the glucose transporter **GLUT1** and increased expression and activity of **G6PD**, in comparison to **CDDP**-sensitive clones (2008). A combination of 6-nicotinamide (**6-AN**) (the **G6PD** inhibitor) and **CDDP** leads to a resensitization of **CDDP**-resistant cells (107). Moreover, since another ovarian cisplatin-resistant cancer cell line, IGROV PT, presented a higher expression of **G6PD**, the same combination has been loaded in liposomes and tested. The results showed a resensitization of resistant cells to cisplatin (138).

The increment in glycolysis is a common characteristic of drug-resistant breast cancer cells independent of the chemotherapeutic treatment, but this augmented activity is regulated in different ways in several resistant breast tumors. It has been demonstrated that triple-negative breast cancer (TNBC) and HER2 positive breast cancer possess higher rate of glycolytic activity than estrogen receptor-positive (ER+) breast cancer cells (139–141). In TNBC, it was shown that EGF pathways are activator of the first step in glycolysis (142) and that MYC modulates this metabolic phenotype by inhibiting the expression of the thioredoxin-interacting protein (143). The different expression of **GLUT** isoforms in breast cancer correlates with tumor cell differentiation, pathological grade, and prognosis. Invasive breast cancer, HER-2 positive, and TNBC, mostly present with a higher glycolysis rate due to the highest expression of **GLUT** (139). The most invasive breast cancer type, TNBC, has the highest **GLUT-1** expression (139). Moreover, overexpression of ErbB2 increased the expression of **LDHA**; promoting glycolysis and breast tumor growth (144). Increased glycolytic rate and a higher sensitivity toward inhibition of glycolysis were demonstrated in lapatinib-resistant BT474 breast cancer cells by a multi-omics approach (125). Curiously, the increase glycolytic activity in BT474 cells was not resulting from an overexpression of glycolytic enzymes, but merely from modifications in the phosphorylation state of glycolytic enzymes, demonstrating that post-translational changes alone can modulate glycolysis. In trastuzumab-resistant ErbB2-positive breast cancer cells, the improved glycolytic rate is regulated by heat shock factor 1 and **LDHA** and inhibition of glycolysis with **2-DG** and the **LDH** inhibitor oxamate by-pass trastuzumab resistance (145). Finally, in paclitaxel-resistant breast cancer cells, synergistic effects on inducing apoptosis were shown in **LDHA** downregulated cells or with oxamate (a pyruvate analog that inhibits the conversion of pyruvate to lactate) association (146). **LDHA** expression and activity are higher in taxol-resistant breast cancer cells. Downregulation of **LDHA** or oxamate treatment resensitizes taxol-resistant cells to taxol (146).

Differently to other tumor cell types that showed a higher rate of glucose consumption early in the modification process, prostate cancer (PCa) cells shift to the Warburg effect only in the metastatic stage, excluding the possibility to use advanced diagnostic procedures like standard FDG-PET scan for the detection of cancer in the early stages (147, 148). Glucose transporters have not been contemplated in PCa evolution because glucose metabolism in the prostate gland is different than in other organs. However, the relevance of **GLUTs** transporters has been lately proposed since the importance of increasing nutrients uptake, comprising glucose is clearly confirmed in PCa. In PCa androgens induce glucose uptake, upregulation of **GLUT** transporters and increased the AMPK pathway (149, 150). Glycolysis varies between androgen-sensitive and insensitive cells and it has been demonstrated that more aggressive tumors showed a higher glucose dependence (151). Indeed, prostate cancer cells switch to aerobic glycolysis only in the metastatic stage (147, 148). Even if the metabolic mechanism that supports prostate cancer metastasis has not been elucidated, in androgen-sensitive cells LNCaP and VCaP, androgen signaling induces both glycolysis and **OxPhos** (152). An augmented activity of key glycolytic enzymes by androgens has been established. **HK2** phosphorylation is prompt by androgens via PKA signaling, while **PFKFB2** is induced by direct binding of androgen receptor (AR) to **PFKFB2** promoter. Activation of **PFKFB2** produces a constitutive activation of 6-phosphofructo-2-kinase 2 (**PFK2**), which is entailed in the second irreversible reaction of the glycolytic pathway (150).

Combining different glycolytic inhibitors with chemotherapeutic agents could be a strategy to overcome drug resistance. To increase the anti-tumor activity **2-DG** was used *in vitro* and *in vivo* in combination with inhibitors of lysosomal permeabilization (153). Moreover, the combined treatment between **2-DG** and fenofibrate (PPARα agonist) caused a synergic effect in cancer cell growth (154). The combination of **2-DG** and paclitaxel in mouse xenografts models of human osteosarcoma and non-small cell lung cancer resulted in a significant reduction in tumor growth (155). **3-BrPa** use *in vivo* conditions resulted in anti-tumor activity after a single injection in a rabbit VX2 hepatoma model (156). Moreover, cells treated with **3-BrPa** enhanced doxorubicin-resistant cells response to the drug (112). Dichloroacetate (**DCA**), a **PDK1** inhibitor, was frequently used in combination with different chemotherapeutics agents (157). The combined treatment of **DCA** with paclitaxel was able to sensitize NSCLC resistant cells (158). Moreover, the combined treatment of **DCA** and **CDDP** was able to decrease tumor growth in advanced bladder cancer (159). The silencing of **PKM2** in lung cancer cells enhanced the efficacy of docetaxel (160). Another group reported that **PDK3** knockdown inhibited hypoxia-induced glycolysis and increased the susceptibility of cancer cells to paclitaxel (161). Cao et al. demonstrated that leukemia daunorubicin-resistant cells show increased expression of GLUT1 and that the combined treatment between daunorubicin and phloretin, an inhibitor of glucose transporter sensitizes K562/Dox cells (113). Doxorubicin-resistant cell lines from anaplastic thyroid cancer presented a high expression of **G6GD** (an enzyme of **PPP**). **G6GD** knockdown or the anthraquinone physcion decreased **G6GD** activity and resensitized doxorubicin-resistant cells (111).

## Targeting Glutamine Metabolism

Tumor cells increase glutamine metabolism to preserve the citric acid cycle, especially given the loss of the entry from pyruvate, in order to adapt to the modifications in the glycolytic pathway (162). Also, glutamine metabolism contributes to cancer cell chemoresistance. Recent studies demonstrated that the use of small molecules, of which several are in clinical trials, to inhibit key enzymes in glutaminolysis pathways is effective in slowing the proliferation of cancer cells (163–168).

Bis-2-(5-phenylacetamido-1,3,4-thiadiazol-2-yl)ethyl sulfide (**BPTES**) was recognized to be the first allosteric inhibitor of **GLS1** (169). It has demonstrated to reduce the proliferation in several cancer cell types *in vitro* and in xenograft models. Unfortunately, its poor metabolic stability and low solubility have discouraged its clinical development (169). For this reason, new inhibitors have been developed, such as **CB-839** that is a more potent inhibitor of **GLS1** than BPTES (170). **CB-839** reduced proliferation of mouse HCC cells at very low concentration, while **BPTES**, at the same concentration, had no activity (171). **CB-839** is ongoing in phase 1 clinical trial for the treatment of various cancer types [Study of the Glutaminase Inhibitor CB-839 in Solid Tumors https://clinicaltrials.gov/ct2/show/NCT02071862]. Glutamine analogs, such as azaserine, acivicin, and 6-diazo-5-oxo-L-norleucine (**DON**), are one strategy to disrupt glutamine metabolic pathways They form covalent bonds with Ser286 in the active site of GLS1 (166). These compounds have demonstrated to block the proliferation of a variety of cancers and have shown their efficacy in some clinical trials (163). Unfortunately, the main problem related to the clinical use of azaserine, acivicin, and **DON** is their lower selectivities toward **GLS1**, since they inhibit other glutamine-dependent enzymes. Then, **compound 968** was identified as an allosteric inhibitor of **GLS1**; and was shown to have cytotoxic effects in lymphoma, breast cancer, glioblastoma, and lung cancer (172–176).

It has been demonstrated that high expression of **GLS** can promote glutamine-independent growth and resistance to therapies that limit glutamine metabolism (177, 178). Thus, other pharmacological approaches to target glutamine metabolism include the use of glutamine synthetase or **GLUD** (Glutamate dehydrogenase) inhibitors (179).

Analysis *in vitro* demonstrated that a high glutamine flux protected MCF7 cells from tamoxifen-induced apoptosis (180). Indeed, a higher content of glutamate was correlated with breast cancer outcomes in patients (181). Metabolomic analysis of 270 breast tumor samples and 97 normal breast samples showed that breast tumor cells had a higher glutamate-to-glutamine ratio than normal cells (182). Another study showed that highly invasive and drug-resistant breast cancer cells presented increased glutamine metabolism, increased glutamate-to-glutamine ratio, and a higher glutaminase expression compared to non-invasive breast cancer cells (172). Moreover, deprivation of glutamine or **BPTES** treatment in combination with **CDDP** or etoposide enhanced chemotherapy cytotoxicity on breast cancer HCC1937 cells (183). Anti-proliferative effects of 1,4-di(5-amino-1,3,4-thiadiazol-2-yl)butane compound, **GLS1** inhibitor, on human breast tumor lines are similar to **BPTES** or **CB-839** (184). Co-treatment of **CB-839** and everolimus interrupts the growth of these endocrine-resistant xenografts (185).

**GLS1** and **GLS2** inhibitors or **BPTES** co-administered with doxorubicin demonstrated a synergistic activity decreasing proliferation of the human pancreas adenocarcinoma ascites metastasis (AsPC-1) cells (186). Disruption of glutamine metabolic pathways improved the efficacy of gemcitabine treatment (nucleoside analog that works by blocking DNA replication) in pancreatic cancer (187).

Some studies have revealed that some invasive ovarian tumor cells improve the use of glutamine to fuel **TCA** cycle (188). Yuan et al. demonstrated that **compound 968** is able to block cell proliferation and sensitize paclitaxel in ovarian cancer (189). Moreover, it has been demonstrated that ovarian cancer **CDDP**-resistant cells present increased glutamine consumption and increased expression of the glutamine transporter **ASCT2** and **GLS**. Thus, the combined treatment of **BPTES** and **CDDP** resensitized **CDDP**-resistant cells (108). Another molecule is epigallocatechin gallate (**EGCG**), which is a **GLUD** inhibitor **GLUD**. This compound combined with **CDDP** had a synergic effect on A2780(cisR) ovarian cancer cells becoming a strategy to overcome cisplatin resistance (190).

mTOR inhibitors-resistant glioblastoma cells have a compensatory increase in glutamine metabolism, suggesting that combined inhibition of **GLS1** and mTOR could potentially overcome resistance (191).

## Targeting Lipid Metabolism

Lipid metabolism is another important player in the development of chemoresistance. The interest in therapeutic strategies directed to block lipid synthesis, lipid uptake, intracellular lipolysis/lipid utilization, and lipid storage is growing (192).

Among the enzyme that regulates lipid metabolism, **FASN** is an important one and it correlates with poor prognosis in various types of cancer and also interferes with drug efficacy (193). Moreover, **FASN** overexpression induces resistance to antitumoral drugs such as adriamycin and mitoxantrone in breast cancer cells (194), gemcitabine-resistant pancreatic cells (195), cisplatin-resistant ovarian cancer cells (110), and radiotherapy resistant head and neck squamous cell carcinomas (196).

Inhibitor compounds targeting lipogenic enzymes (such as **FASN**, **ACLY**, and **ACC**) have been studied and their anticancer activity has been demonstrated in several preclinical models (197–199). Besides the promising data, serious side effects of these compounds have led to their clinical development exclusion. Various **FASN** inhibitors, such as Cerulenin, C75, orlistat, C93, C247, and GSK837149A, have been identified (200). The inhibition of **FASN** demonstrated to represent an excellent target, when used in *in vitro*, in xenograft and genetically induced mouse model analysis (200). Inhibitors of **FASN** induced cancer cells death directly or sensitized them to chemotherapic drugs, such as 5-fluorouracil and trastuzumab (201–204).

It has been reported, by genomic profiling, that **CPT1** and fatty acid input into an oxidative pathway are decreased in four aggressive cancer cells, including melanoma, breast, ovarian, and prostate malignancies, respect to their non-aggressive counterparts (205). Recent studies reported a negative correlation between **FASN** inhibition and the consequent effect on body weight and food intake. In fact, a worsen eating that leads to weight loss was observed in mice treated with cerulenin and C75 and the cause seemed to be related to the **CPT**-1 inhibition in the hypothalamus (206–208).

The **SPHK1** sphingosine Kinase 1 isozyme has been largely studied and its several functions in tumor development have been demonstrated, while the **SPHK2** has not been as well-studied (209–213). Several studies *in vitro* and *in vivo* (only preclinical) demonstrated that ABC294640, the **SPHK2**-specific inhibitor, is able to inhibit proliferation of cancer cells or tumors more effectively or similarly than agents targeting **SPHK1**, in several tumor models, including ovarian (214), multiple myeloma (215), lung (216), kidney (217), breast (218), prostate (219), and pancreatic cancers (220).

Liver X receptor (LXR) is a crucial transcriptional regulator of cholesterol homeostasis and SR9243 is an LXR inverse agonist. SR9243 is able to kill selectively cancer cells because it inhibits the Warburg effect and lipogenesis and so the inhibition leads to the formation of an environment not favorable to cancer cells (221).

Recently, several studies have shown that dysregulated sphingolipid metabolism is a key contributor to the progression and resistance of ovarian cancer. By RNA-seq, Dobbin and colleagues revealed transcriptional variants between matched pairs of carboplatin and paclitaxel-treated vs. control patient-derived xenograft (PDX) models of ovarian cancer. In particular, they identified that S1P signaling is modified pathways following chemotherapy treatment (222). Sphingolipid metabolizing enzymes strictly related in modulating the ceramide-sphingosine-S1P rheostat play a key role in cell proliferation and have been directly correlated with drug resistance in ovarian cancer (223, 224). Specifically, increased expression of ceramide transport protein (**CERT**), **SPHK1**, **SPHK2**, and glucosylceramide synthase (**GCS**) have been correlated with resistance to paclitaxel, doxorubicin, and N-(4-hydroxylphenyl) retinamide (fenretinide) chemotherapies and cytotoxicity (225–230). The sphingolipid-mediated sphingosine-1-phosphate (**S1P**) pathway may represent a promising new pharmacological target to counteract the chemoresistance in ovarian cancer cells. Few therapeutic compounds specifically target **S1P** pathway proteins, but this pathway can modify the response of several chemotherapeutic treatments, including docetaxel, doxorubicin, and cyclophosphamide (231–234). Several approaches have been studied for modulating sphingolipid metabolism, and some of them consist in the use of combined treatment between ceramide analogs and chemotherapeutic agents (235–237). Treatments that showed activity in resistance ovarian cancer models include the use of synthetic ceramide analogs, inhibitors of **SPHK**, neutralization of secreted **S1P**, and **S1PR** antagonists. For example, the combined treatment of C6-ceramide with paclitaxel showed a synergic effect in suppressing cell proliferation and migration of CAOV3 ovarian cancer cells (238, 239). Moreover, drug delivery systems seem to be useful. In fact, a resensitization to paclitaxel of taxane-resistant SKOV3.TR ovarian cancer cells have been shown with the combination of paclitaxel with C6-ceramide-encapsulated in poly(ethylene oxide)-modified poly(epsilon-caprolactone) (PEO-PCL) nanoparticles (235). Kelly M. and colleagues demonstrated that the combined treatment of tamoxifen with the Sphingosine kinase 1 (**SK1**) inhibitor FTY720 blocks proliferation of both ERα-positive and ERα-negative drug-resistant cell lines and an ERα-positive PDX model of ovarian tumor (240). The multiple mechanisms of action of tamoxifen and its relatively high therapeutic index provide a strong rationale for combining tamoxifen with FTY720, as a strategy for treating ovarian tumors and circumventing drug resistance (226, 241–243).

It has been demonstrated that tamoxifen-resistant breast cells, T-47D, present an increased level of neutral lipids, in particular, cholesterol esters and triglycerides, and increased expression of Peroxisome Proliferator-Activated Receptor Gamma (PPARγ) (121). **CDDP**-resistant human ovarian cancer cell lines shift their metabolism toward a lipogenic phenotype and accumulate lipid droplets (244). Moreover, **CDDP**-resistant lung cells have an increased expression of **FASN** and that inhibiting **FASN** could decrease the metastatic potential of **CDDP**-resistant cells (245). It has been demonstrated that a combination of orlistat, an inhibitor of **FASN** and cisplatin, *in vivo*, causes a delay in tumor growth (110). A high fatty acid synthase (**FASN**) activity is also involved in ErbB2-induced breast cancer chemoresistance to docetaxel (116). It has been demonstrated that prostate-resistant cells, C4-2R and MR49F (enzalutamide-resistant cells) respect to C4-2 and LNCaP have increased expression of 3-hydroxy-3-methyl-glutaryl-coenzyme A reductase (**HMGCR**). They showed that the combined treatment between simvastatin and enzalutamide sensitizes resistant cells *in vitro*. Moreover, tests *in vivo* in xenografts mice demonstrate a decrease in tumor cell proliferation (122).

Inhibitors of **CPT1**, such as etomoxir or ranolazine, have demonstrated promising results in different types of tumors. In fact, the combined treatment of etomoxir and orlistat is able to inhibit cell proliferation in LnCaP and VCaP prostate cancer cells (246). Moreover, the treatment of human leukemia cells with etomoxir or ranolazine can induce apoptosis cell death (115).

## Targeting Mitochondria Metabolism

During cancer cells' adaptation to an hypoxic microenvironment, mitochondria have been demonstrated to be fundamental during solid tumor metastasis and in chemoresistance (247–249). Targeting mitochondrial-dependent metabolism to overcome drug resistance is an area of intense research. The increase of antioxidant pathways that help cancer cells to neutralize mitochondrial **ROS** is a common strategy adopted by some tumors to become drug-resistant (250). Moreover, mitochondria could promote therapy resistance by reducing the mitochondrial permeability transition (**MPT**) and inducing apoptosis resistance (251).

Mitochondria also appear responsible for the accumulation of oncometabolites such as fumarate, succinate, and 2-hydroxyglutarate (**2-HG**). Indeed, Succinate dehydrogenase complex iron-sulfur subunit B (**SDHB**), fumarate hydratase (**FH**), isocitrate dehydrogenase [NADP(+)] 1, cytosolic (**IDH1**), isocitrate dehydrogenase [NADP(+)] 2 and mitochondrial (**IDH2**) may be affected by germline or somatic mutations in a variety of human tumors (252). Fumarate, succinate and **2-HG** accumulation is sufficient to drive malignant transformation and thus behave like *bona fide* oncometabolite (253). These oncometabolites share the capacity to inhibit α-ketoglutarate-dependent enzymes that control gene expression at the epigenetic level, such as Jumonji domain (JMJ) histone lysine demethylases (254) as well as ten-eleven translocation (TET) dioxygenases (255), resulting in the expression of a potentially oncogenic transcriptional program associated with a block in terminal differentiation (256).

Dysregulation of mitophagy (removing of abnormal mitochondria) contributes to neoplastic progression and drug resistance in various tumors (257). Enhanced mitophagy can protect cancer cells during chemotherapy and radiotherapy preventing apoptosis (258). On the other hand, excessive mitochondrial clearance may induce metabolic disorders and cell death (259). Therefore, mitophagy likely plays a dual role in cancer drug resistance (260). Mitophagy inhibition enhances the sensitivity of breast cancer cells to classical paclitaxel (114).

mtDNA has an essential role on tumorigenesis and chemoresistance. mtDNA pathogenic point mutations and changes in copy number, were shown to induce cancer progression (261) and have been associated with cancer development to a more malignant phenotype with poor prognosis *in vivo* (262–264). However, most mtDNA mutations are neutral missense mutations present in homoplasmy (265), suggesting that severe mutations are negatively selected. Indeed, mtDNA mutations *per se* are not able to induce carcinogenesis (266) but some mtDNA polymorphisms correlated with tumor development (267–270).

In particular, mutations in mitochondria encoded Complex I (CI) subunits could affect tumor progression depending on their mutational load and its detrimental activity (271). Based on these observations, Gasparre et al. introduced the concept of oncojanus: severe CI assembly mutations can promote tumorigenesis below a threshold level; but above that level they behave as antitumorigenic due to CI assembly defects. On the other side, non-disassembling mild mtDNA CI mutations could stimulate tumor proliferation and metastases. The oncojanus function of CI subunits was described for both mitochondrial and nuclear encoded CI subunits (272, 273). CI disruption inhibits OxPhos, promote NADH accumulation, inhibition of α-ketoglutarate dehydrogenase and increase the α-Ketoglutarate (KG)/succinate ratio. The α-Ketoglutarate (KG)/succinate imbalance activates prolyl-hydroxylases (PDH) enzymes responsible for the hydroxylation and degradation of HIF-1α even in hypoxic conditions (271, 274). Of note, genetical and pharmacological targeting of CI activity in osteosarcoma and colorectal cancer cell models successfully converted a carcinoma into a benign low-proliferating and non-invasive oncocytic tumor (273).

Moreover, the oncojanus effect was also observed in ovarian cancer after chemotherapy (275). A missense mtDNA point mutation in the MTND4 subunit of CI appeared after carboplatin treatment and generated a mild energetic defect allowing paclitaxel chemoresistance. When mutated MTND4 arrived to a certain threshold CI activity was impaired turning cancer cells into an oncocytic phenotype. Later it was demonstrated that the accumulation of deleterious mtDNA mutations induced by carboplatin in ovarian cancer cell lines conferred paclitaxel resistance through the reduction of filamentous tubulin (276). The treatment of A549 non-small cell lung cancer cells with CDDP induced an homoplasmic shift of a non-synonymous mutation in the CI protein MTND2 resulting in chemoresistance to cisplatin; which was correlated with the upregulation of the nuclear PGC-1α and PGC-1β and increased mitochondrial biogenesis (277).

mtDNA depletion in cancer cells under drug treatment promotes invasion and metastasis, induces expression of epithelial-to-mesenchymal (EMT) proteins (278) and activates pro-survival and antiapoptotic pathways (279, 280). Although the detailed molecular mechanism remains to be determined, several studies have demonstrated that reduced mtDNA content promotes activation of a mitochondria-to-nucleus signaling leading to increased expression of anti-apoptotic genes, including Bcl-2, and activation of pro-survival enzymes, such as Akt (280), that likely play a role in conferring resistance to apoptosis induced by drug treatment. mtDNA depletion in androgen-dependent LNCaP prostate cancer cells resulted in the loss of androgen dependence and increased resistance to paclitaxel (118, 119).

Horizontal transfer of mtDNA to cancer cells with a low respiratory function was correlated with recovery of respiration and increased tumor-initiating efficacy (281). mtDNA exchange through intercellular bridges or exosomes (extracellular vesicles implicated in cell-cell communication and transmission of disease states) could induce drug resistance by promoting a cancer stem cell (CSC) phenotype (282) Interestingly, exosomes containing mtDNA and mitochondrial proteins involved in mitochondrial fusion and biogenesis were found in the serum of prostate cancer patients as well as in the tumor itself (283). MSCs also protected AML leukemia cells from chemotherapeutic cytotoxicity by transferring them functional mitochondria. These effects occur together with mitochondrial fragmentation controlled by ERK-mediated Drp1 phosphorylation. Thus, disruption of leukemia cells/stromal interactions and targeting mitochondrial dynamics may provide a novel strategy that could be combined with conventional chemotherapeutic agents for leukemia treatment (284). It was demonstrated that the co-culture of leukemia cells with mesenchymal stem cells (MSCs) increased the expression of uncoupling protein 2 (**UCP2**) in leukemia cells; uncoupling oxidative phosphorylation and decreasing **ROS** production (285, 286). Recent studies have demonstrated the protective role of mitochondrial metabolism on cancer cells exposed to chemotherapy cytotoxicity. In acute myeloid leukemia (AML) adenosine 5′-monophosphate (AMP)-activated protein kinase (**AMPK**) signaling promoted glucose uptake and a shift to glycolysis decreasing intracellular ROS (287).

Phenformin is a biguanide similar to metformin that targets complex I of mitochondria. It was identified to be more potent in decreasing cell growth in non-small cell lung cancer, but unfortunately drug-resistance emerged (288). It has been hypothezed that the development of resistance is dependent on functional LKB1-**AMPK** signaling, which improves a switch in their metabolism to bypass inhibitory effects of phenformin. Since the serious side effects of the drug, it was withdrawned from the market. Besides this, new studies on phenformin have been conducted (288).

**PGC-1α** is a transcription co-activator that regulates mitochondria biogenesis and it is involved in energy metabolism. It has been reported that **CDDP** treatment increases **PGC-1α** levels in a small cell lung carcinoma cell line and **PGC-1α** silencing sensitizes cells to this drug (109). Increased mitochondrial mass confers stem-like properties to breast cancer cell lines MDA-MB-231 and MCF7 and enables their resistance to paclitaxel (117). **PGC-1α** induction may also cause chemoresistance by activating a metabolic shift to bypass **ATP** request, as shown for cells treated with inhibitors of BRAF (289). This is also confirmed for 5-FU resistance, which increased **PGC-1α** expression and so modified cellular cancer metabolism to modulate the energetic stress induced by treatment (290–292).

Numerous emerging studies demonstrated a correlation between mitochondrial dynamics and cell survival (293–298). The co-culture of leukemia cells with MSCs also altered the mitochondrial dynamics of leukemia cells due to an increase of the activating phosphorylation of Dynamin-1-like protein (Drp1) at S616. Drp1 is a GTPase that regulates mitochondrial fission. Leukemia cells overexpressing wild-type Drp1 or Drp1 S616E presented fragmented mitochondria, reduced mitochondrial **ROS** levels, increased glycolysis, and improved drug resistance (299–301). Drp1 S616 phosphorylation through the stimulation of mitochondria fission and glycolysis seemed required to RAS-induced transformation (302). Moreover, inhibition of Drp1 activity caused mitochondrial fusion and impeded tumor growth (303). The cell cycle inhibitor Cytarabine is a normally chemotherapeutic therapy for leukemias and lymphomas, with reduced clinical implications due to the development of resistance (304, 305). Among the probable mechanisms, Cytarabine treatment improved the increase of a chemoresistant leukemic stem cell population with high **FAO**/**OxPhos** activity. In light of this, the **FAO** inhibitor etomoxir blocks oxygen consumption in acute myeloid leukemia cells and sensitized cells to cytarabine (306).

Recent evidence suggests that chemoresistant ovarian cancer has an increase in **OxPhos** dependence. Improved **OxPhos** in ovarian cancer cells increase IL-6 production (307) which facilitates tumor cell survival and proliferation (308), changing efficacy to chemotherapy, and reduce progression-free survival of ovarian cancer patients (309). Ovarian cancer cell migration was shown to be sustained by pyruvate, involving the mitochondrial activity during metastasis (310). Other studies have revealed that some invasive ovarian tumor cells improve the use of glutamine to fuel the **TCA** cycle (188). CD44+CD117+ ovarian tumor stem cells derived from epithelial ovarian cancer patients exhibited both high glucose uptake and a high **OxPhos** phenotype, which was correlated with their amplified capacity to live under a glucose-free context (311). On the other hand, CD44+MyD88+ cancer stem cells had a mainly glycolytic phenotype and suggested that therapy with glycolytic inhibitors could be favorable to increase patient's survival (312). Mouse ovarian cancer-initiating cells (putative cancer stem cells) harbor a highly flexible metabolic phenotype, whereby they could use either glycolysis or **OxPhos** under stress (313). It was proposed that most ovarian tumor cells may use either glycolysis or OXPHOS and that such plasticity increased their “cellular fitness” (310, 314, 315). The shift from glycolysis to **OxPhos** has also been showed upon MYC/KRAS or MYC/ERBB2 removal in breast cancer cells (316, 317), and also in glioma cells because of the acquired resistance to phosphoinositide-3-kinase (PI3K) (318). Moreover, PI3K resistance in breast cancer cells is related to the shift from glucose to lactate (34). Inhibitors of mitochondrial respiration become therapeutic strategies in ovarian cancer cells because of their dependence on **OxPhos**. In fact, cancer-selective inhibition of the electron transport chain (**ETC**) could kill ovarian cancer cells directly without affecting normal cells. The complex I inhibitor BAY 87-2243 has been studied in a Phase 1 study (NCT01297530) but results were not showed (Clinicaltrials.gov). Several strategies targeting mitochondria CIII complex, such as the thiol modifier β-phenylethylisothiocyanates (**PEITC**), have been developed. In particular, **PEITC** can enhance the **ROS** level, decreasing **OxPhos** and, consequently, causing prostate cancer cell death by apoptosis (319). Moreover, the same compound combined with metformin demonstrated cytotoxicity in human ovarian cancer cells (320). Also, treatment with ABT-737, the inhibitor of Bcl-2 proteins (321) and the FAO inhibitor perhexiline also was capable of sensitized **CDDP**-resistant ovarian cancer cells (322) by modulating mitochondrial metabolism. The main drugs to target mitochondria or disrupt **OxPhos** are antibiotic or anti-parasitic agents. Among these, azithromycin and doxycycline target mitochondrial protein synthesis, while salinomycin targets mitochondrial K+/H+ exchange (323). Azithromycin, combined with **CDDP** and paclitaxel, is able to reduce side effects and to enhance patients' survival (324). Doxycycline is able to inhibit cellular growth of ovarian cells SKOV3 and SKOV3/DPP and to sensitize resistant cells to **CDDP** (325). Salinomycin is another antibiotic able to inhibit cell growth, especially on cancer cells compared to normal epithelial cells (326).

Resistance to 5-FU has been associated to aumgmented mitochondrial mass and activity, increase **ETC** enzymes expression and higher level of oxygen consumption (290, 327). So, due to their **OxPhos**-dependency, resistant cells were showed to be sensitive to Complex I inhibition by metformin (327). **OxPhos** involvement to 5-FU resistance was correlated to the development of stemness-related phenotype, stricly linking CSCs to mitochondrial metabolism as described previously (328, 329). According, the activation of mitochondrial **FAO** is able to promote stemness in gastric cancer cells and consequently there is chemoresistance to 5-FU induced by tumor-associated mesenchymal stem cells. In fact, treatment with the **FAO** inhibitor etomoxir was capable to partially reduce FU resistance (330).

BRAFV600E is a mutation found in stage IIIc or stage IV melanoma. Chapman and co-workers demonstrated that its inhibition with vemurafenib leads to the shift to **OxPhos** and the switch is useful to treat resistant melanoma cells (331). Elesclomol sodium (STA-4783) is a compound targeting **ROS** in tumor cells. Its mechanism is strictly related to inhibition of electron transport flux and so increase **ROS** generation causing oxidative stress in both malignant and healthy cells. However, as cancer cells have already higher **ROS** production, this drug will be capable to cause cytotoxicity selectively in malignant cells, resulting in activation of apoptosis in melanoma cancer cells (332). STA-4783 alone and in association with paclitaxel revealed promising results in phase I/II clinical studies in patients with refractory solid tumors (333, 334), but unfortunately serious side effects lead to stop phase III study in melanoma patients (335).

Lee and coworkers demonstrated that an enhanced mitochondrial oxidative phosphorylation characterizes irinotecan-resistant NSCLC cells. They tested a combined treatment between gossypol (a molecule that inhibits aldehyde dehydrogenase) and phenformin (a molecule that inhibits mitochondrial complex I), concluding that the combination leads to sensitization of irinotecan-resistant NSCLC cells (336). Another drug targeting mitochondria is apogossypol, which is semisynthetic. It has been demonstrated to be cytotoxic in murine B cells (337). Metformin, a drug usually used for the treatment of type 2 diabetes, has demonstrated anti-cancer properties. In fact, the combined treatment of metformin and paclitaxel showed anticancer activity *in vivo* and was able to arrest the cell cycle *in vitro* in human breast MCF-7 and human lung A459 cancer cells (338). PC3 prostate cancer cells docetaxel-resistant shift their metabolism from glycolysis toward OXPHOS and this is linked to EMT phenotype. The combination of chemotherapy and OXPHOS inhibitors limited docetaxel-associated drug resistance and progression toward metastasis (120).

In short, targeting glycolysis, **PPP**, **OxPhos**, and fatty acid synthesis and oxidation could enhance chemotherapy and radiation responsiveness and overcome therapy resistance. Importantly, therapy-resistant tumors present different metabolic phenotypes related to non-treated tumors, thus it's needed a better understanding of the new dependencies and vulnerabilities across different chemotherapy treatments in different tumors to reduce toxicity and to increase the efficacy of chemotherapeutic drugs.

## Concluding Remarks

Metabolic deregulation is an established hallmark of cancer, thus the elucidation of novel therapy combinations based on new tumor-specific metabolic liabilities after chemotherapy will be essential to the development rational metabolic therapeutic strategies to overcome drug resistance.

## Author Contributions

MD, IG, TP-G, and MM wrote the manuscript.

### Conflict of Interest

The authors declare that the research was conducted in the absence of any commercial or financial relationships that could be construed as a potential conflict of interest.
